# Effect of sample density in prompt γ-ray analysis

**DOI:** 10.1038/s41598-022-08594-2

**Published:** 2022-06-01

**Authors:** Makoto Maeda, Mariko Segawa, Yosuke Toh, Shunsuke Endo, Shoji Nakamura, Atsushi Kimura

**Affiliations:** Nuclear Science and Engineering Center, Japan Atomic Energy Agency, 2-4 Shirakata, Tokai-mura, Naka-gun, Ibaraki 319-1195 Japan

**Keywords:** Experimental nuclear physics, Characterization and analytical techniques

## Abstract

A high-accuracy analytical method is broadly required to obtain reliable research results. Thus, prompt γ-ray analysis (PGA), one of the most accurate non-destructive analytical methods, has been employed in various fields. However, the measurement accuracy of PGA is also known to degrade in hydrogenous samples. The degradation is caused by variation in the measurement sensitivity (counts per milligram) following the change in neutron energy due to scattering with hydrogen nucleus. Number of scatterings is well known to depend on the hydrogen content in a sample. However, considering multiple scatterings, hydrogen density, which has not been taken into account as yet, may also lead to the accuracy degradation. Here, we show the effect of the hydrogen density in PGA by evaluating the measurement sensitivity of samples with the same hydrogen content and different densities. We find that the measurement sensitivity varies by more than 30% depending on the hydrogen density even at the same hydrogen content. The variation is a particularly serious problem for PGA requiring a few percent accuracy in most cases. Additionally, although the variation is apparently observed in hydrogenous samples, the similar phenomenon can occur in other nuclides with a large scattering cross section; it may affect nuclear cross-section measurements using neutrons in such fields as astrophysics and nuclear energy.

## Introduction

In prompt γ-ray analysis (PGA), a sample is irradiated with neutrons to excite the nuclei intended to be measured, and the γ-rays emitted during the de-excitation processes are measured to quantify the amount of target nucleus in the sample. Although thermal neutrons are generally used, some measurement systems utilize cold neutrons, which most nuclides have a larger capture cross section than the thermal one, to achieve more efficient anaysis^1,2^. PGA is a powerful analytical method that has high measurement accuracy, enables us to non-destructive analysis of bulk samples, and can be applied to light elements which are difficult to measure by most other analytical methods^3–5^. Owing to the aforementioned advantages, PGA can be applied in many fields such as measurements of cadmium in rice^6^, measurement of boron in volcanic rock^7^, elemental analysis of meteorites^8,9^, and measurement of hydrogen in metal samples by focusing on hydrogen embrittlement in the industrial field^10^.

Although PGA can measure light elements such as hydrogen, it suffers from a problem in which the measurement accuracy of hydrogenous materials degrades. The rate of γ-ray emission, denoted as *R*_g_ can be expressed as follows,

1 $$ R_{{\text{g}}} \propto {\text{N}} \times \varphi \left( E \right) \times \sigma \left( E \right), $$

where N is the number of nuclei, *φ(E)* is the neutron flux, and *σ(E)* is the neutron capture cross section. Equation (1) represents the rate of nuclear reactions that emit γ rays such as (n, g) and (n, α). Equation (1) shows that measurement sensitivity, which is defined by the γ-ray counts per number of nuclei in PGA, strongly depends on the energy distribution of neutron flux (or neutron energy spectrum) in the sample. One of the causes of measurement-accuracy degradation is neutron scattering in the sample. The scattered neutrons change their flight path and length in the sample, and the neutron flux apparently varies depending on the sample geometry. Thus, the measurement accuracy is degraded with the variation in the measurement sensitivity. (This phenomenon is called as “effect of apparent neutron flux”). Mackey et al.^11–13^ minimized the accuracy degradation by suppressing the change in the path lengths using spherical samples.

**Figure 1:**
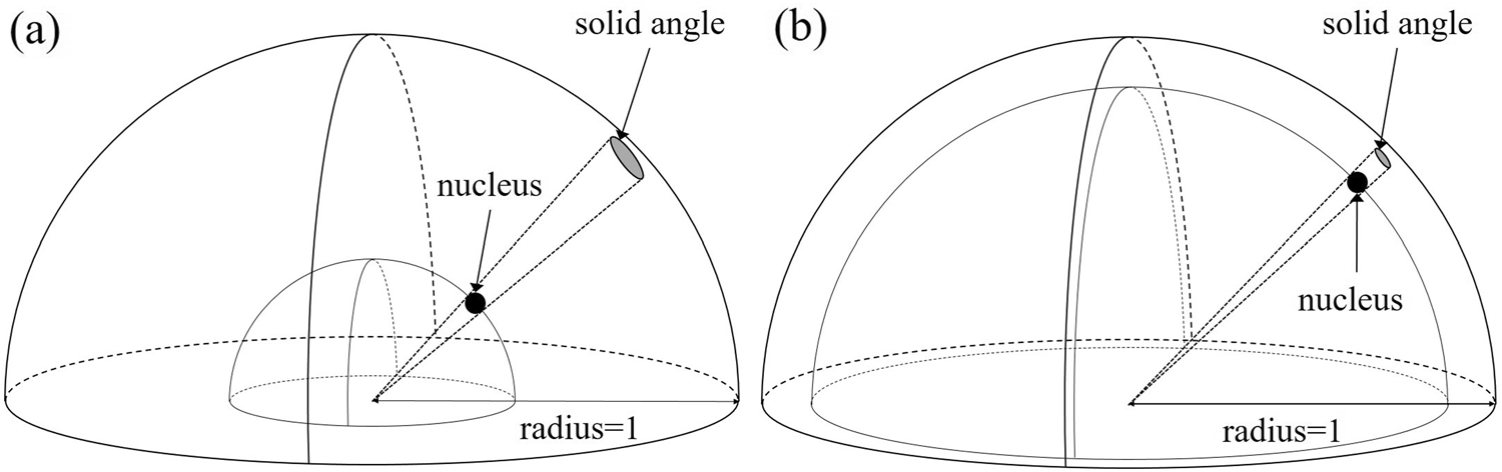
Comparison of the solid angles that contribute to the probability of the second or later neutron scattering reaction between (**a**) high- and (**b**) low- density samples.

Paul et al. reported that in PGA that uses cold neutrons, sensitivity is affected not only by the effect of apparent neutron flux but also by the change in neutron energy in scattering with hydrogen nucleus. To obtain precise analysis results, the sensitivity should be calibrated as a function of the target geometry and scattering power, which depends on the probability of neutron scattering and amount of energy change of neutrons during scattering^14,15^. By considering the same way as Eq. (1) for the scattering reaction, the scattering power depends only on the number of hydrogen nucleus, or hydrogen content, in the sample. In PGA that uses cold neutrons, accurate analysis results can seem to be obtained by suppressing the effect of apparent neutron flux using spherical samples and applying a correction which depends on the hydrogen content. However, this process may be insufficient, as explained hereunder. A large difference exists between the scattering and (n, γ) reactions depending on whether the neutron remains or disappears due to absorption after the reactions. The remaining neutron can scatter again after the first scattering, that is, multiple scatterings can occur. Although the probability of the first scattering obeys Eq. (1), the probability of the second or later scattering will depend on the solid angle from the scattered neutron to the next nucleus. In this case, since the solid angle varies with the internuclear distance which depends on the sample density, the number of scatterings depends not only on the hydrogen content but also on the hydrogen density. Here, the hydrogen density refers to how densely hydrogen nuclei exist in the sample. Figure 1 shows schematic views of the solid angles in (a) high- and (b) low- density samples. The solid angle is defined by a projected area on the surface of a unit sphere. When a neutron is scattered by a nucleus at the center of a hemisphere, the high-density sample has a larger solid angle with a small distance between the nuclei. On the other hand, the low-density sample has a smaller solid angle with a larger distance between the nuclei. Therefore, the probability of scattering, which depends on the solid angle, is also larger for higher density samples. Thus, the sensitivity is affected by the hydrogen density as well as the hydrogen content. This phenomenon occurs not only in hydrogen but also in all nuclides with large scattering cross sections. However, hydrogen is the most important element used in many fields, present in various samples, and an element that can be measured by PGA with superior results compared with other analytical methods. This is why we focus on hydrogen in this study. Since the neutron energy change during scatterings occurs other than the thermal energy region, accuracy also degrades in PGA that uses epithermal neutrons. In the present research, we have quantitatively evaluated the effect of hydrogen content and density on PGA that uses cold and epithermal neutrons by Monte Carlo simulation and validated the simulation results by experiments.

## Results

### Effect of hydrogen content on sensitivity

First, to confirm that the sensitivity of PGA changes with the hydrogen content instead of the effect of apparent neutron flux, the sensitivity was evaluated by simulation for different hydrogen contents using samples of the same density and different sizes. In simulation, Monte Carlo simulation code PHITS^16^ was used, along with nuclear-data library JENDL4.0^17^. As mentioned in “Introduction” section, spherical samples were used to suppress the effect of apparent neutron flux. Polystyrene (C_2_H_4_)_n_ samples with a density of 1.05 g/cm^3^ and diameters of 6, 8, 10, and 12 mm, were irradiated with mono-energetic neutron beams with a diameter of 22 mm and energies of 25 meV (thermal neutron), 5 meV (cold neutron), and 1 eV (epithermal neutron). The sensitivities were evaluated by counting the 2223 keV γ-rays emitted in a neutron capture reaction of hydrogen.

**Figure 2:**
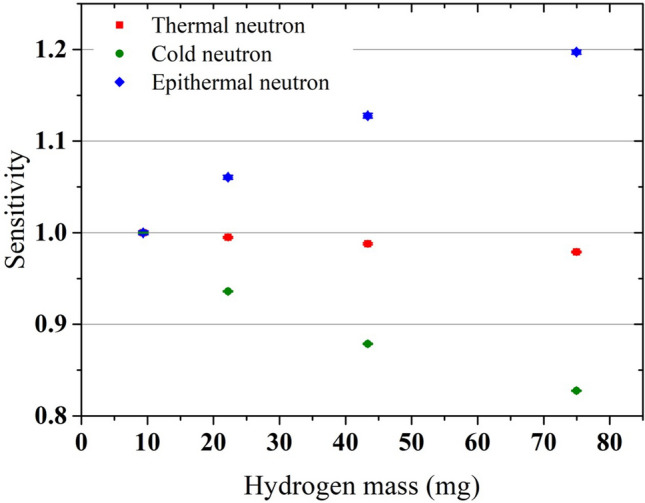
Evaluation results of the sensitivities of polystyrene samples in the thermal-, cold-, and epithermal- neutron irradiations. The sensitivities are normalized by those of the 6 mm-diameter samples for each incident-neutron energy. The error bars reflect one sigma standard deviations.

Figure 2 shows the evaluation results of the sensitivities normalized by those of 6 mm- diameter samples at each incident-neutron energy. In the thermal-neutron irradiation, the sensitivities were almost constant, similar to those reported by Mackey et al. In the cold- and epithermal-neutron irradiations, the sensitivities decreased by up to 17% and increased by up to 20%, respectively, with the increase in the hydrogen content. Using the mass attenuation coefficient^18^, an attenuation of 2223 keV γ-rays in the sample was calculated to be 3.0% for the largest sample and 1.5% for the smallest sample. It indicates that the differences in sensitivities were mainly caused by the differences in the probabilities of the capture reaction rather than the attenuation of γ-rays. These results can be explained by the following. In the cold-neutron irradiation, because the energy of the thermal motion of the hydrogen nucleus was larger than that of the incident-neutron, the contribution of up-scattering, in which the neutron receives energy during scattering, was large. This effect increased the neutron energy and decreased the sensitivity because of the smaller cross sections at higher neutron energy. On the other hand, in the epithermal-neutron irradiation, the sensitivity increased due to the contribution of down-scattering, in which the neutron loses energy during scattering. In both cases, as the hydrogen content increased, the number of scattered neutrons increased. Therefore, the energy change in the neutron flux also increased and the degree of sensitivity change became larger.

### Effect of hydrogen density on sensitivity

Next, the effect of hydrogen density on the sensitivity was evaluated. In the evaluation, simulation was performed using PHITS under the condition where only the density and size of the samples were changed from that presented in the previous section. The density was varied by 1/1 (density of polystyrene alone: 1.05 g/cm^3^), 1/2, 1/5, 1/10, and 1/50.

**Figure 3:**
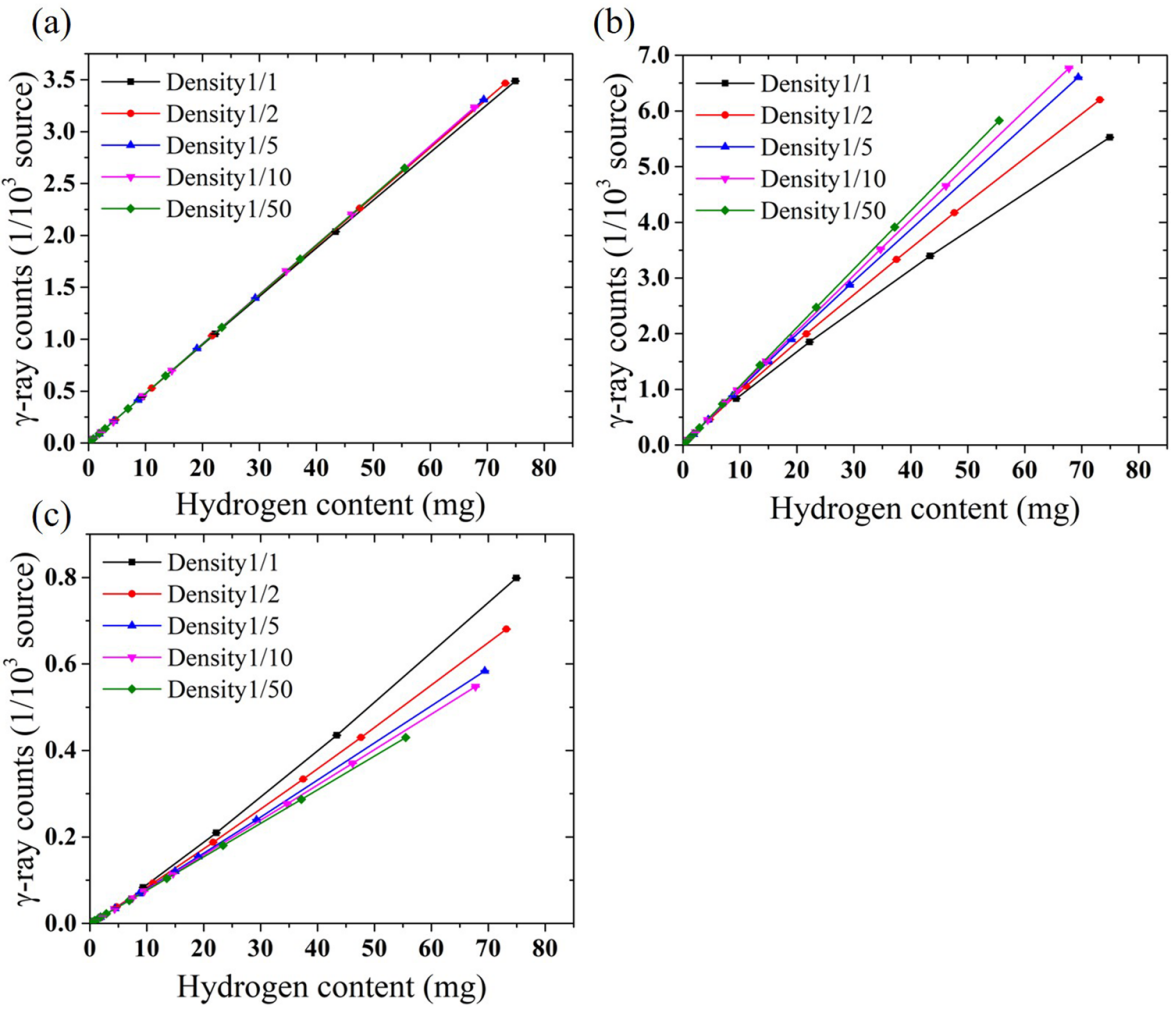
Correlations between γ-ray counts and hydrogen content under each sample density in the (**a**) thermal-, (**b**) cold-, and (**c**) epithermal- neutron irradiations.

**Figure 4:**
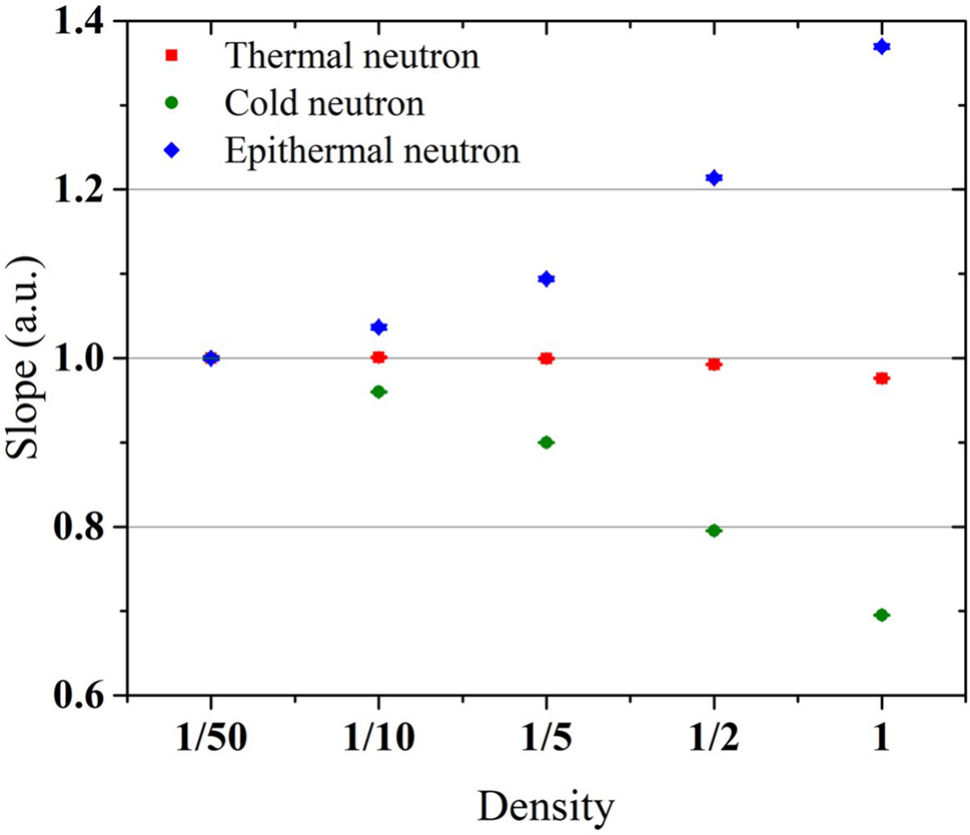
Slopes of the correlation graphs shown in Fig. 3 obtained by linear fit for each neutron energy and sample density. The slope has the same dimension as the sensitivity (counts per hydrogen mass). The slopes were normalized by those of the sample with a density of 1/50. The horizontal axis shows the sample density normalized by that of polystyrene alone. The error bars reflect one sigma standard deviations.

The correlations between the γ-ray counts and hydrogen content in the thermal-, cold-, and epithermal-neutron irradiations are shown in Fig. 3. In the thermal-neutron irradiation, the correlation graphs almost overlapped even at different sample densities since the energy change of the neutrons in scatterings was negligible and the sensitivity was constant, similar to that presented in the previous section. On the other hand, in the cold- and epithermal- neutron irradiations, different γ-ray counts were obtained depending on the densities even at the same hydrogen content. These phenomena indicate that the amount of energy change in the neutron flux due to scattering depends on the density as well as the hydrogen content. To quantitatively evaluate the sensitivity change, the slopes of the correlation graphs shown in Fig. 3 which have the same dimension as the sensitivity (counts per hydrogen content) were derived by linear fit. The obtained slopes for each neutron energy and density are shown in Fig. 4. The results were normalized by those of the sample with a density of 1/50 in which the effect of the energy change due to neutron scattering was the smallest in each neutron energy. In the thermal-neutron irradiation, the slopes were almost constant at different densities since the energy change was negligible. In the cold- and epithermal-neutron irradiations, as the density increased, the slopes decreased by up to 30% and increased by up to 37%, respectively. As mentioned in the previous section, the γ-ray attenuation was 3.0% for the heaviest sample with 1/1 density and 12 mm-diameter and the differences in the sensitivities were mainly caused by the neutron energy. The number of scatterings increased with the density because the distance between the nuclei became smaller and the solid angle became larger. As a result, the amount of energy change in the neutron flux also increased. Thus the degree of sensitivity changes increased. The degree of sensitivity changes further increases when a higher density sample is measured or incident neutron energy is lower than 5 meV or higher than 1 eV. We confirmed that the hydrogen density, as well as the hydrogen content, significantly affects the measurement sensitivity of PGA that uses cold and epithermal neutrons.

### Validation of the simulation results

Finally, to validate the simulation results, polystyrene samples were measured in the accurate neutron-nucleus reaction measurement instrument (ANNRI) installed at the Materials and Life Science Experimental Facility in the Japan Proton Accelerator Research Complex^19–21^. In the ANNRI, PGA with a broad neutron-energy range can be performed, while the incident-neutron energy is measured using the time-of-flight (TOF) method, which is named TOF-PGA. In other words, the γ-ray spectrum for each incident-neutron energy can be obtained. In the validation, different from the previous two sections, the samples with diameters of 10, 13, 15, and 20 mm with densities of approximately 1/5 and 1/10 were used. Detailed explanation is shown in Method section.

**Figure 5:**
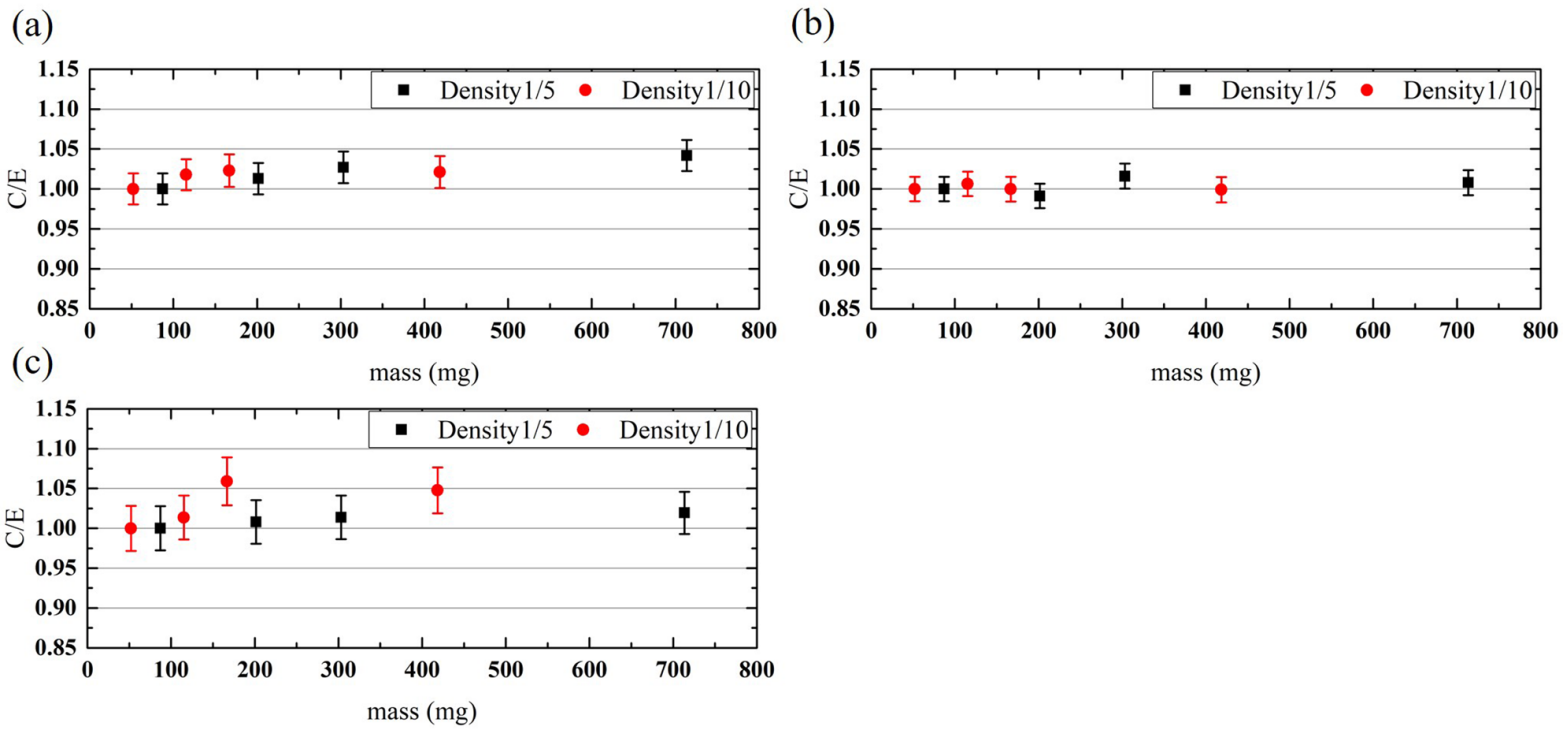
Comparison of the experimental and simulated results of the sensitivities for the (**a**) thermal-, (**b**) cold-, and (**c**) epithermal-neutron irradiations. The C/E value is defined as the ratio of the simulation and experimental results. The error bars reflect one sigma standard deviations.

Figure 5 shows the C/E values, which are defined as the ratio of the calculation results obtained by simulation to the experimental data, of the sensitivities for each sample and neutron energy. The C/E values were between 0.99 and 1.06. In general, simulations reproduced the experimental results well and were confirmed to be valid.

## Discussions

### Effect of neutron scatterings

To confirm that the cause of the sensitivity change is the difference in the number of scatterings, which depends on the sample density, the number of scatterings is formulated using approximation. We compare the neutron-energy spectra in the samples with different densities, which are designed to produce the same number of scatterings using the derived equation.

**Figure 6:**
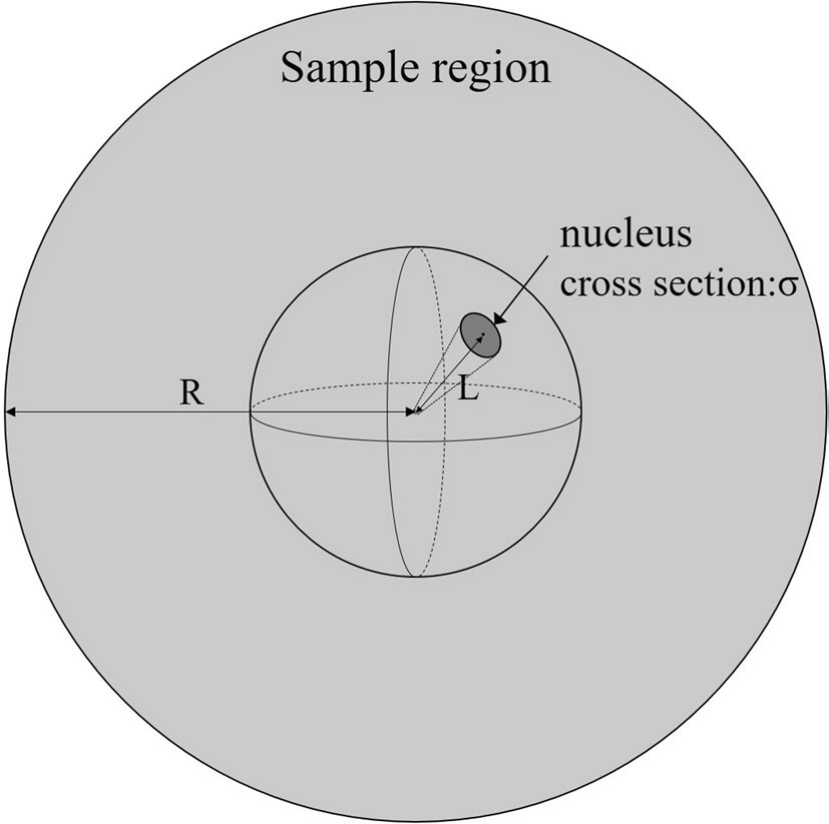
Schematic diagram of a spherical sample.

Here, we consider a spherical sample with scattering cross section *σ*, distance between the nearest nuclei *L*, sample radius *R*, and density *ρ*, as shown in Fig. 6. Assuming that the neutron is isotropically scattered, expected value *N* of the number of second or later scatterings can be expressed as a product of the scattering probability, which depends on the solid angle, and the path length of the neutron in the sample.

2 $$ N = \frac{{{\text{C}}\sigma }}{{4{\uppi }L^{2} }}\frac{{{\text{K}}R}}{2L} = \frac{{{\text{CK}}R\sigma }}{{8{\uppi }L^{3} }} $$

where C is a constant and K is a function that depends on the incident position and flight direction angle of the neutron.

Next, we consider matching the number of scatterings in two samples that consist of the same nuclei with scattering cross section *σ*. One of the samples has density *ρ*_1_, radius *R*_1_, and distance between nuclei *L*_1_ while the other has density *ρ*_2_, radius *R*_2_, and distance between nuclei *L*_2_. When the numbers of scatterings in both samples match, we obtain

3 $$ \frac{{{\text{CK}}R_{1} \sigma }}{{8\pi L_{1}^{3} }} = \frac{{{\text{CK}}R_{2} \sigma }}{{8\pi L_{2}^{3} }}. $$

Since the samples are assumed to have the same elemental composition and only differ in density, the number of the nearest nuclei is the same, i.e.,

4 $$ \frac{{4{\uppi }L_{1}^{3} }}{3}\rho_{1} = \frac{{4{\uppi }L_{2}^{3} }}{3}\rho_{2} . $$

From Eqs. (3) and (4),

5 $$ \frac{{\rho_{1} }}{{\rho_{2} }} = \frac{{R_{2} }}{{R_{1} }}. $$

According to Eq. (5), the numbers of scatterings of the samples with different densities can be matched by creating a radius ratio that is equal to the inverse ratio of the densities. When the numbers of scatterings are matched, the measurement sensitivities as well as the neutron energy spectra in both samples will be equal.

**Figure 7:**
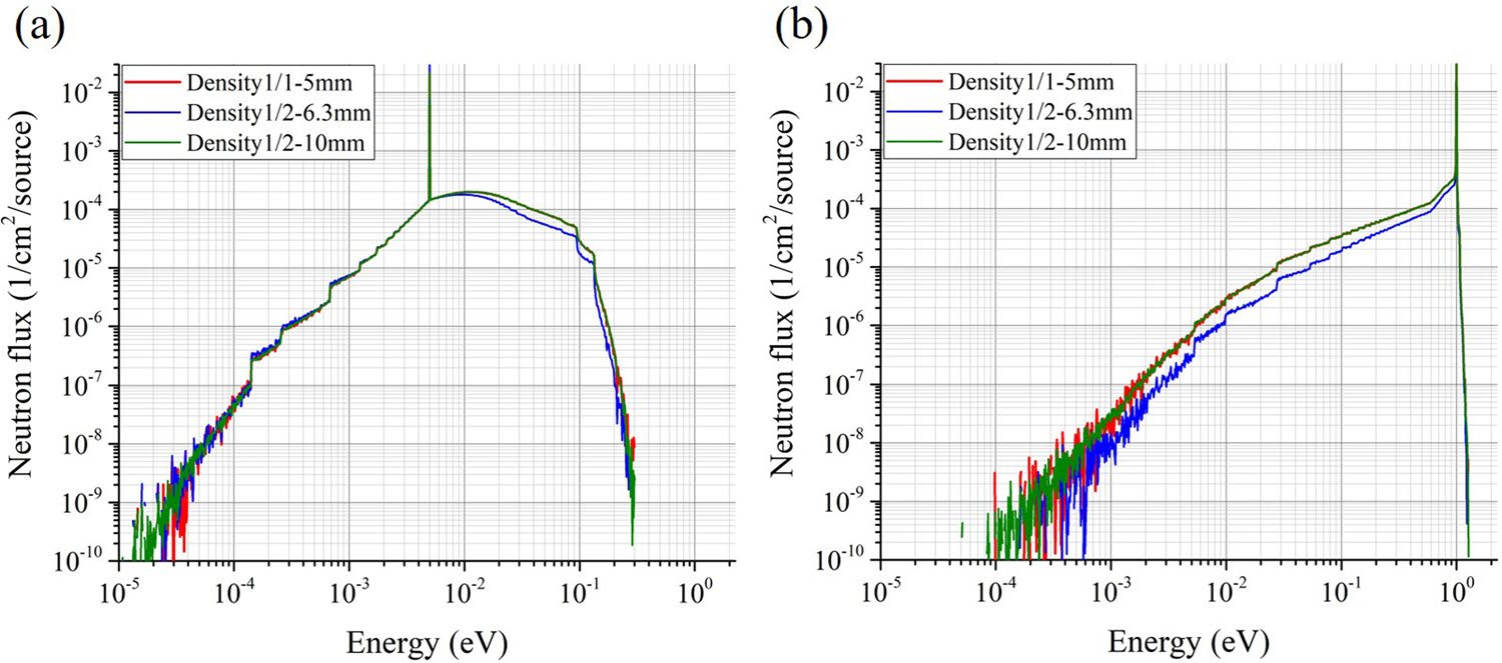
Neutron-energy spectra in the samples for the (**a**) cold- and (**b**) epithermal-neutron irradiation. The density is normalized value to that of polystyrene alone. The sample with a density of 1/1 and radius of 5 mm is a standard sample. The hydrogen content of the sample with a density of 1/2 and radius of 6.3 mm is matched with that of the standard one. The number of scatterings of the sample with a density of 1/2 and radius of 10 mm is matched that of the standard one.

For verification, the neutron energy spectra were simulated using PHITS under the same condition as that presented in the previous section for the cold and epithermal neutrons incident on three polystyrene spherical samples. The first was a standard sample with a density of 1/1 (density of polystyrene alone: 1.05 g/cm^3^), and a radius of 5 mm. The second was a sample with a density of 1/2 and a radius of 6.3 mm and matched the hydrogen content to the standard sample. The third was a sample with a density of 1/2 and a radius of 10 mm and matched the number of scatterings to the standard sample. Figure 7 shows the obtained spectra for each sample under the (a) cold- and (b) epithermal- neutron irradiations. Peaks at 5 meV in (a) and 1 eV in (b) were made by incident neutrons without scatterings. The spectrum of the second sample (the masses matched) is different from that of the standard sample, whereas the spectrum of the third sample (the numbers of scatterings matched) agrees well with that of the standard sample under both irradiations. These results indicate that the sensitivity change in the samples with different densities is mainly caused by the difference in the numbers of scatterings in the sample. Further, almost the same neutron energy spectra and measurement sensitivities can be obtained from the samples with different densities by matching the number of scatterings, which satisfies Eq. (5).

Although the apparent effect of the density is observed in the analysis of a sample that contains hydrogen, which has a large scattering cross section, this phenomenon can intrinsically occur in other nuclides. Therefore, even for samples without hydrogen, the effect needs to be taken into account in cold- and epithermal- neutron experiments such as PGA that uses cold neutrons and neutron cross-section measurements, which are important in the astrophysics and nuclear-energy fields. In addition, re-evaluation of the nuclear data based on past measurements that did not take into account the effect of density may be necessary.

## Summary

In this research, we evaluated the effects of the hydrogen content and the hydrogen density on PGA that uses cold and epithermal neutrons by simulations, and validated the simulation results by TOF-PGA experiments. The results revealed the importance of the effect of the hydrogen density on PGA as well as the hydrogen content. In simulation, we confirmed that the measurement sensitivities varied by more than 30% depending on the hydrogen density. For different sample densities and incident-neutron energies, the sensitivity will vary by more than this value. The sensitivity change was caused by the variation in the neutron spectrum in the sample due to the neutron scattering with hydrogen nucleus. Even if the prepared samples had the same hydrogen content, the sensitivity change was observed because of the variation in the number of scatterings, which depends on the hydrogen density. Although this effect is apparently observed in a sample with hydrogen, it can also intrinsically occur in other nuclides.

This effect is not restricted to TOF-PGA in ANNRI. The same effect cannot be neglected in measurement facilities that use neutrons other than those in the thermal-energy region. The sample density affects the experiments in many fields, for example, in PGA that uses cold and epithermal neutron and in astrophysics and nuclear energy that require nuclear cross section data. Re-evaluation of the nuclear data may be required if the density effect is not considered in the analysis of nuclear-data experiments.

## Methods

### Experiments in ANRRI

TOF-PGA was performed by utilizing the high-intensity pulsed neutron source and high-efficiency cluster high purity germanium (HPGe) detectors installed in ANNRI. The measurements were performed using a beam diameter of 22 mm, a flight path of 21.5 m, two cluster HPGe detectors, four coaxial HPGe detectors, and a helium atmosphere in the sample space. Spherical polystyrene samples with a density of approximately 1/5 of a simple-substance polystyrene and diameters of 10, 13, 15, and 20 mm (weights of 87.3, 201.6, 303.3, and 713.6 mg, respectively) and the samples with density of approximately 1/10 that of a simple-substance polystyrene and diameters of 10, 13, 15, and 20 mm (weights of 52.1, 115.3, 166.8, and 418.5 mg, respectively) were measured. The samples were double-wrapped in a 25-µm-thick FEP film and set on a Teflon sample holder. The samples were measured with a beam power of 600 kW for 2–14 h, depending on the hydrogen content of the sample, to within a statistical error of 3% in epithermal-neutron irradiations.

In the analysis, the energy regions of the cold, thermal, and epithermal neutron were set at 4.77–5.22 meV, 24.2–26.8 meV, and 0.8–1.3 eV, respectively. In the γ-ray spectrum obtained by gating in each incident neutron energy region, the capture γ-ray peak of hydrogen at 2223 keV was analyzed to evaluate the sensitivity.

## Data Availability

The data that support the plots within this article and other findings of this study are available from the corresponding authors on reasonable request.

## Code availability

PHITS code is available at https://phits.jaea.go.jp/howtoget.html.
